# Unwelcome Immigrants: Sources of Opposition to Different Immigrant Groups Among Europeans

**DOI:** 10.3389/fsoc.2019.00024

**Published:** 2019-04-12

**Authors:** Anastasia Gorodzeisky, Moshe Semyonov

**Affiliations:** Department of Sociology and Anthropology, Tel Aviv University, Tel Aviv, Israel

**Keywords:** european immigration, attitudes toward immigrants, exclusion, public opinion, ethnic groups

## Abstract

The present paper advances the proposition that level of opposition to immigration (i.e., endorsement of closure or exclusion) and its sources are not uniform and vary across immigrant groups. To test this proposition we utilize data from the 2014 European Social Survey for 20 countries and apply the analysis to the following groups: immigrants of same race/ethnic group as a majority population, immigrants of different race/ethnic group, Muslim, Jewish, and Roma immigrants. The analysis reveals that level of opposition to immigration of different ethno-religious groups in Europe is hierarchical, being most extreme toward Muslims and Roma and quite minor toward people of the same ethnic/race groups as well as toward Jews. Further analysis reveals that not only the level of opposition varies across groups but also the sources that drive such opposition. In general, the sources of opposition to immigration can be divided to 2 major categories: universal sources and group-specific sources. The universal sources (sources which increase opposition toward all immigrants regardless of their origin) pertain to threat of competition over socio-economic and symbolic resources. The group-specific sources consist of racism, fear of crime, and inter-group contact. Racism and lack of inter-group contact tend to increase opposition that is exclusive to Muslim and to Roma immigrants. Racism, however, does not increase opposition that is exclusive to immigrants belonging to a race/ethnicity, which is different from most country people. Fear of crime is likely to prompt opposition that is exclusive to immigrants of different race/ethnic group and to Roma but not toward Muslims. The findings underscore the multiple sources underlying emergence of anti-immigrant sentiment, in general, and opposition to specific groups of immigrants, in particular.

## Introduction

Exclusionary policies have long been understood along the Weberian theoretical concept “closure” according to which “social collectivities seek to maximize rewards by restricting accesses to resources and opportunity to a limited circle of eligible” (Parkin, 1974, p. 44). From this perspective, researchers have long viewed opposition to immigration as a form of closure resulting from fear of competition over rewards and resources, whether real or symbolic (Quillian, 1995; Fetzer, 2000; Scheepers et al., 2002; Semyonov et al., 2006). In the present paper, we seek to contribute to the literature on formation of attitudes toward immigrants by advancing the thesis that opposition to immigration is not unidimensional but is prompted and motivated by multiple sources. We further contend that the level of opposition to immigration and its sources are not uniform across all groups of immigrants. We suggest that whereas opposition to immigration, regardless of immigrants' religious and ethnic origin, tend to increase with fear of competition over socio-economic and symbolic resources and to decrease with intergroup contact, racist views, and fear of crime prompt opposition to immigration directed at specific ethnic and religious groups.

To date the overwhelming majority of studies on anti-immigrant sentiments have examined attitudes toward immigrants as a generic category, not distinguishing between groups by ethnic origin and by religion. This approach can be problematic because members of the public may have in mind different types of immigrants when asked to report their views on a general category of immigrants (Blinder, 2015). The small and quite recent body of research that distinguishes between groups of immigrants by ethnic or religious origin found that in the European context attitudes toward immigrants vary across groups being more negative toward ethnic minorities (Gorodzeisky and Semyonov, 2009; Ford, 2011; Ben-Nun Bloom et al., 2015); and that opposition is especially pronounced in the case of Muslim (Strabac and Listhaug, 2008; Hellwig and Sinno, 2017) and Roma out-group populations (Fontanella et al., 2016).

To put to test the theoretical arguments that opposition to immigration is multi-dimensional and that the level and sources of opposition to immigration vary across ethno-religious groups, we utilize data from the European Social Survey (gathered in 2014 from 20 countries). In the data analysis, we estimate and compare levels and sources of opposition to immigration across several immigrant groups (i.e., immigrants of same race/ethnic group as a majority population, immigrants of different race/ethnic group, Muslim, Jewish, and Roma immigrants). By discussing the meaning of the findings in light of sociological theory we seek to provide a broader and deeper understanding of the multiple sources underlying emergence of anti-immigrant sentiment, in general and opposition to specific groups of immigrants, in particular.

## Sources of Opposition to Immigration

The “competitive threat” theoretical model is the theoretical framework most often used by social scientists for understanding emergence of negative attitudes toward out-group populations. According to the model, anti-immigrant sentiment (including prejudicial views and exclusionary attitudes) should be understood as a reaction to threat of competition (whether real or perceived) with immigrants either in the economic sphere (e.g., labor market, welfare system) or in the cultural sphere (e.g., cultural homogeneity of a society; social values). From this point of view, the “competitive threat” theoretical perspective is unidimensional and as such it provides a theoretical framework that does not allow inclusion of additional sources of anti-immigrant sentiments. However, overview of previous studies on anti-immigrant attitudes reveals several additional sources that play a role in the formation of attitudes toward out-group populations but do not originate from fear of competition. For example, a substantial body of socio-psychological research focuses on intergroup social contact that affects attitudes toward outgroup via interactions (e.g., Pettigrew and Tropp, 2006). Likewise, a few recent studies suggest that racial prejudice (as beliefs that acquired via socialization) and fear of crime (which does not stem from threat of competition) affect exclusionary attitudes toward immigrants (e.g., McLaren and Johnson, 2007; Gorodzeisky and Semyonov, 2016). Although competitive threat, intergroup contact, racist views, and fear of crime are somewhat interrelated, each can constitute a distinct source of anti-immigrant sentiment. Therefore, we contend here, that intergroup contact, racist beliefs, and fear of crime are independent of “competitive threat” and each represents a distinct and unique determinant of anti-immigrant sentiment.

To advance the knowledge on the sources of opposition to immigration, the present study endorses a model which includes multiple sources of opposition to immigration. Hence, it examines the unique contribution of each one of the following four major sources: threat of competition, (lack of) intergroup contact, fear of crime, and racist views. In what follow, we discuss the above-mentioned sources of opposition to immigration in detail and then conclude with expectations related to the relevance of each source to the formation of opposition toward immigrants in general and toward specific ethno-religious groups of immigrants in particular.

### Competitive Threat in the Economic Sphere

The competitive threat theoretical model (also known as group threat) operates under the premise that intergroup relations are shaped by group identification coupled with intergroup competition over rewards and resources (e.g., Blumer, 1958; Blalock, 1967; Bobo and Hutchings, 1996). The intergroup competition is defined in terms of a zero-sum game with asymmetric power relations between the competing groups. Members of the majority population view themselves as superior to the out-group populations and therefore more deserving access to privileges, resources, and rewards (Blumer, 1958; Bobo and Hutchings, 1996). Therefore, when an out-group population (e.g., immigrants) poses a challenge (whether real or perceived) to the privileges and interests of the majority group in socio-economic sphere, hostility and exclusionary attitudes toward the others are likely to rise. From this point of view, opposition to immigration can be understood as a defensive reaction toward emerging threats and challenges posed by members of the out-group population to the superiority of the majority population in access to social and economic resources (See support to this argument by e.g., Scheepers et al., 2002; McLaren, 2003; Raijman et al., 2003; Semyonov et al., 2004). Following this logic, we expect economic threat to increase opposition to immigration of all groups of immigrants.

### Competitive Threat in the Cultural Sphere

The second source of exclusionary attitudes is driven by perceptions of threat posed by the out-group population to the cultural homogeneity and the national identity of the host society (Fetzer, 2000; Raijman and Semyonov, 2004; Sniderman et al., 2004; Gorodzeisky, 2013). According to this view, members of the majority population, regardless of threat to their economic interests, might be concerned with the impact that the out-group population exerts on the national and cultural character of the host society. More specifically, some members of the majority group are often disturbed with the detrimental impact that outsiders may exert on the national culture, collective identity, value-system and homogeneity of the national population (e.g., Schnapper, 1994; Fetzer, 2000; Castles et al., 2014). In other words, members of the majority population object to immigrants because they fear that immigrants “pollute” the local culture and the homogeneous composition of the national population (e.g., Ben-Nun Bloom et al., 2015). Following this argument, we expect fear of competition in the cultural sphere, regardless of threat to economic interests, to increase opposition to immigration of all groups of immigrants.

### Intergroup Contact

Intergroup contact is viewed as a major source of positive attitudes toward out-group populations (e.g., Allport, 1954; Pettigrew, 1998; Brown and Hewstone, 2005; Semyonov and Glikman, 2008). Engagement with members of an out-group population, especially when the contact is positive, decreases prejudice and hostility toward the out-group population. On the other hand, lack of contact is likely to preserve prejudice and negative attitudes toward out-group populations (Allport, 1954; Pettigrew, 1998; Brown and Hewstone, 2005). That is, intergroup contact can alter attitudes and beliefs about the others through intimate personal experience, deeper knowledge, affective ties, and in-group reappraisal. It occurs via process of generalization of positive attitudes from the encountered member of an outgroup to the outgroup and affective processes of reduced intergroup anxiety and threat perceptions (Hewstone, 2015). The theory further suggests that inter-group friendship has the strongest effect on eliminating negative attitudes and prejudice (Pettigrew, 1998). Moreover, not only do positive attitudes emerge toward a specific outgroup with which contact was established but the positive attitudes seem to permeate and spread toward other outgroups as well (Hewstone, 2015). Although the causal relations between contact and attitudes are not fully established, a large body of research lends firm support to the thesis that contact is likely to decrease negative attitudes and reduce hostility toward the outgroup populations (for meta-analysis see Pettigrew and Tropp, 2006). Following the logic embodied in the contact theoretical model, we expect intergroup contact to decrease opposition to immigration of all groups of immigrants.

### Fear of Crime

One of the widespread beliefs held by members of the majority population regarding the detrimental impact of immigrants on the social environment is the idea that immigrants are responsible for rise in crime and violence (Calavita, 2003; Semyonov et al., 2006, 2008; Ceobanu, 2011). According to Ceobanu (2011; p. 126), for example, Europeans' concerns of immigrants' impact on crime “are perhaps reinforced by the fact that some immigrants come illegally or overstay their visa.” A large body of research has repeatedly revealed that fear of crime is among the major reasons why native-born do not want to share residential space with ethnic minorities and immigrants; and that fear of crime and lack of sense of personal safety are more pronounced in residential areas where racial minorities and immigrants are highly concentrated (Semyonov et al., 2012, for Europe). Indeed, fear of crime committed by immigrants, has become one of the major sources of opposition to immigration in the European context (McLaren and Johnson, 2007; Turper, 2017)^1^. Following these studies, we expect fear of crime to increase opposition to immigration mostly in the case of (visible) ethno-religious minorities (the immigrants that are most often perceived as associated with criminal activities).

### Racism and Prejudice

A series of studies carried out in the European context emphasize the central role played by racial/ethnic prejudice in shaping attitudes toward immigrants (e.g., Pettigrew and Meertens, 1995; Pettigrew, 1998; Verberk et al., 2002; Vala et al., 2008; Gorodzeisky and Semyonov, 2016). While some view racial prejudice as resulting from competitive threat (e.g., Verberk et al., 2002), others contend that racist views constitute an independent source of anti-immigrant sentiments. For example, a recent study by Gorodzeisky and Semyonov (2016) demonstrates that racial prejudice toward the non-European/non-white minority population is likely to increase negative attitudes toward immigrants, regardless of competitive threat.

Racial prejudice is defined as “antipathy based on faulty and inflexible generalization” (Allport, 1954, p. 9). It was traditionally viewed as socially learned feelings, sentiments, and cultural ideas (Allport, 1954; Kinder and Sears, 1981; Sears and Kinder, 1985). In other words, racial prejudice is an irrational socially acquired feeling with scant economic or social basis. The impact of racial/ethnic prejudice on opposition to immigration may reflect a form of racism. Although racism is strongly associated with racial prejudice, the two concepts do not completely overlap. While racial/ethnic prejudice is defined as negative feeling toward a socially defined group and toward any person perceived to be a member of that group, racism refers to a general ideology and belief in hierarchical order of racial and ethnic groups together with the idea that inherent differences among the racial and ethnic groups determine cultural and individual achievement (e.g., Van den Berghe, 1967). Racism is especially relevant with regards to emergence of opposition toward immigrants belonging to ethnic and racial minorities. This is so because racism is also viewed as the organizational map that guides actions of racial actors in society (Bonilla-Silva, 1997). Following this logic, we expect racist views to increase opposition to immigrants belonging to ethno-religious minorities in host countries, regardless of threat of competition and fear of crime.

In sum, the present paper aims to identify sources that drive opposition to several specific ethno-religious immigrant groups in Europe. Subsequently, in the analysis that follows we will introduce a methodological approach that enables us to isolate and discern the opposition to immigration that is directed “**exclusively”** at a specific group of immigrants from the “**general objection”** to immigration (or from other groups). Then we will proceed to examine the impact of the various sources on opposition to immigration in general and to “exclusive opposition” directed at specific groups of immigrants. By doing so, we will be in a position to test theoretical expectations regarding differential sources of exclusionary attitudes toward various ethnic and religious immigrant groups in Europe.

## Materials and Methods

Data for the present analysis were obtained from the seventh round of the European Social Survey (ESS) 2014 that included “Immigration” module (for detailed information on the “Immigration” module see European Social Survey, 2015). We used information provided by the 2014 ESS on twenty European countries. For each country, data were gathered from a random probability national sample of the eligible resident populations aged 15 and over. The analysis reported here was restricted to the native-born citizens whose parents were born in the country (majority group population).

### Measured Indicators of the Predictors of Opposition to Immigration

***Perceived economic threat***is an index constructed as the mean score of responses to the three following questions: (1) “Would you say that it is generally bad or good for [country]'s economy that people come to live here from other countries?” (2) “Would you say that people who come to live here generally take jobs away from workers in [country], or generally help to create new jobs?”, and (3) “Most people who come to live here work and pay taxes. They also use health and welfare services. On balance, do you think people who come here take out more than they put in or put in more than they take out?” Responses were recoded according to an 11-point scale ranging between 0 (good for the economy, create new jobs, and generally put in more, respectively) and 10 (bad for the economy, take jobs away and generally take out more, respectively). ***Perceived*** ***cultural threat*** is captured by an index constructed as the mean score of responses to the two following questions: (1) “Would you say that [country]'s cultural life is generally undermined or enriched by people coming to live here from other countries?” and (2) “Do you think the religious beliefs and practices in [country] are generally undermined or enriched by people coming to live here from other countries?” Responses were recoded according to an 11-point scale ranging between 0 (enriched) and 10 (undermined). ***Intergroup contact*** is a dummy variable that distinguishes between respondents that have close friends of a different race or ethnic group from most [country] people and those who do not have such friends. Note that positive contacts with members of one outgroup (e.g., different race or ethnic group) are expected to reduce negative attitudes also toward other out-groups (e.g., Muslims, Roma). ***Fear of crime*** is measured by responses to the following single question: “Are [country]'s crime problems made worse or better by people coming to live here from other countries.” Responses were recoded according to an 11-point scale ranging between 0 (better) and 10 (worse). ***Racism*** is operationalized by an index based on respondents' answers to the three following questions: “Do you think some races or ethnic groups are born less intelligent than others?,” “Do you think some races or ethnic groups are born harder working than others?,” “Thinking about the world today, would you say that some cultures are much better than others?” Because responses to these questions were coded only as “yes” and “no,” the variable is expressed as the proportion of the questions that elicited a positive answer (out of all the questions on which a respondent provided answers)^2^.

In order to control for individuals' socio-demographic characteristics, the following variables were used in the estimation procedure: age (in years), gender, education (years of formal schooling), and reported subjective income (insufficient vs. sufficient).

### Measuring Opposition to Immigration: Definitions and Descriptive Overview

The measured indicators of opposition to immigration were obtained from responses to the following five questions: (1) “To what extent do you think [country] should allow people of the same race or ethnic group as most [country] people to come and live here?” (2) “How about people of a different race or ethnic group from most [country]?” (3) “To what extent do you think [country] should allow Jewish people to come and live here?” (4) “To what extent do you think [country] should allow Muslims to come and live here?”, and (5) “To what extent do you think [country] should allow Gypsies to come and live here?” Response options were 1 (many), 2 (some), 3 (a few), and 4 (none). In order to provide the most extreme and clear-cut categories of opposition to immigration, we distinguished between those who said allow “NONE” and all others (response options include: many, some and a few). In Table 1, we present percent distribution of the respondents who object to immigration (“allow none”) by ethno-religious groups of immigrants and by country.

**Table 1:** Percent of respondents who oppose to immigration of a group (allow *NON* from this group to come and live here)…(%), ordered according to the level of opposition.

	**Same race/ethnic group**	**Jewish people from other countries**	**Different race/ethnic group**	**Muslims from other countries**	**Gypsies from other countries**	***N***
Austria	7.7	12.7	15.3	23.5	28.3	1,417
Belgium	8.1	10.7	13.6	20.5	31.9	1,338
Switzerland	1.5	6.0	4.4	14.9	20.5	898
Germany	1.3	2.3	3.7	6.7	12.6	2,457
Denmark	2.0	2.6	6.0	11.3	25.9	1,304
Spain	8.8	12.7	12.5	22.9	29.6	174
Finland	2.5	5.3	8.7	17.9	23.3	1,945
France	6.7	7.2	12.4	14.3	20.4	1,420
United Kingdom	10.2	7.0	14.4	19.4	31.6	1,743
Ireland	9.4	10.9	14.3	25.5	45.1	1,963
Netherlands	5.3	4.1	6.5	14.6	17.3	1,576
Norway	0.8	2.4	1.3	8.3	17.8	1,193
Portugal	13	29.2	18.8	35.5	46.4	1,120
Sweden	0.4	0.9	0.5	3.8	5.0	1,414
Czech Republic	16.8	17.9	29	56.5	63.5	1,891
Estonia	4.2	12	12.2	41.8	51.2	1,133
Hungary	12.4	35.2	32.6	56.3	66.1	1,623
Lithuania	7.6	20.9	12	38.6	50.3	1,967
Poland	6.5	14.2	10.6	34.4	28.7	1,518
Slovenia	6.5	17.0	11.4	22.9	34.0	1,002
Europe	6.5	9.2	11.1	20.2	26.4	30,636

The data reveal that the level of opposition to immigration varies considerably across groups and across countries. Scandinavian countries are characterized by relatively low level of opposition to immigration while Eastern European countries are characterized by relatively high level of opposition to immigration (regardless of the origin of the immigrant group). At the same time, there is a clear hierarchical order in the level of opposition toward the groups with almost uniform order in *all European countries*. Opposition is least pronounced toward “immigrants of a same race or ethnic group as most country people” and most pronounced toward Roma immigrants. Although opposition to Muslim immigrants is lower than that toward Roma immigrants, it is considerably higher than the level of opposition toward “immigrants of a different race and ethnic group” and toward Jewish immigrants. Opposition to Jewish immigrants is higher than that expressed toward immigrants of a same race and ethnic group but lower than that toward immigrants of a different race and ethnic group, with several exceptions^3^.

Specific information regarding the average level of objection (percent of those who checked the “allow none” option) toward the various groups of immigrants in Europe as a whole can be obtained from the values listed in the bottom row of Table 1. It is interesting to note that opposition to immigration (i.e., those not willing to admit any immigrants from specific groups) is quite moderate. Specifically, only 6.5 percent of Europeans object immigration of people of the same race or ethnic group as those living in the country, 9.2 percent object to Jewish immigrants and 11.1 percent object to immigrants of a different race or ethnic group. The percent of opposition to Muslim and Roma immigrants are considerably higher than those expressed toward any of the other groups (20.2 and 26.4, respectively). Indeed, the values in Table 1 attest to the hierarchical order of the level of opposition toward different groups of immigrants with immigrants of the same race/ethnicity being “most welcome” and Muslims and Roma being “least welcome”.

The hierarchical order of opposition to groups of immigrants (that are apparent in Table 1) leads us to expect that those who object to immigration of one group of immigrants are likely to object to other groups. Furthermore, it is reasonable to expect that those who oppose immigration of the “more welcomed” groups are likely to also oppose immigration of the “less welcomed” groups. To put this expectation to test, we display (in Table 2) the overlap between categories of opposition to different groups of immigrants. The findings lend firm support to this expectation. For example, more than 90 percent of respondents who oppose immigrants of a same race and ethnic group object to the admission of any immigrants of different race or ethnicity. Three quarters of those who oppose immigrants of a different race or ethnic group (from most country people) also oppose Muslim and Roma immigrants. More than 90 percent of respondents who object to any Jewish immigrants also object to admission of any Muslim immigrants. Likewise, almost 80 percent of respondents who oppose to immigration of any Muslims also oppose to immigration of any Roma people.

**Table 2:** Percent of those who oppose to immigration (allow none) of each group out of those who oppose to immigration of a certain group.

	**Opposition to immigration of same race/ethnic group** ** (allow none)**	**Opposition to immigration of** ** Jews (allow none)**	**Opposition to immigration of different race/ethnic group** ** (allow none)**	**Opposition to immigration of** ** Muslims (allow none)**	**Opposition to immigration of** ** Gypsies (allow none)**
**Oppose immigration of…**.					
Same race/ethnic group	–	41	54	26	20
Jewish people from other countries	58	–	48	43	32
Different race/ethnic group	92	58	–	41	33
Muslims from other countries	80	93	75	–	61
Gypsies from other countries	80	89	77	79	–

The overlap in opposition to different categories of immigrants makes it difficult to isolate the unique sources that drive opposition toward a specific group. Yet, it is possible that sources that drive opposition to Muslim immigrants (or opposition to immigrants of different race or ethnicity) are different from the sources that drive opposition to immigrants of the same race or ethnicity or Roma. Therefore, it is important to identify and isolate respondents who oppose **to only** one ethno-religious group of immigrants from those who object immigration of all groups or from those who are willing to admit all groups of immigrants. Along the same line of logic, it is also important to identify and isolate respondents who are willing to admit **only** immigrants of the same race or ethnic group but exclude all others. To overcome such identification problems, we constructed a set of mutually exclusive categories of opposition (or admission) to immigrants by ethno-religious origin. The classification scheme resulted in seven categories of respondent's attitudes regarding admission of the various groups of immigrants to the country:

1. *Pro-admission* includes all respondents who do not object to any of the five ethno-religious groups (i.e., willing to admit a few, some or many immigrants).
2. *Total exclusionists* pertain to respondents who oppose to all five ethno-religious groups (by stating “allow none to come and live here” regarding all groups).
3. *Exclusive admission of the same race/ethnic group* consists of respondents, who do not object to immigrants of the same race or ethnic group but object to immigrants from all other ethno-religious groups (i.e., different race/ethnic group, Jewish people, Muslims, and Roma).
4. *Exclusive opposition to a different race/ethnic group* includes respondents who object immigrants of a different race or ethnic group but willing to admit immigrants belonging to all other four ethno-religious groups (i.e., same race/ethnic group, Jewish people, Muslims, and Roma).
5. *Exclusive opposition to Jewish people* contains respondents who object Jewish immigrants but willing to admit immigrants belonging to all other four ethno-religious groups (i.e., same race/ethnic group, different race/ethnic group, Muslims and Roma).
6. *Exclusive opposition to Muslims* includes respondents who object to Muslim immigrants but willing to admit immigrants belonging to all other four ethno-religious groups (i.e., same race/ethnic group, different race/ethnic group, Jewish people, and Roma).
7. *Exclusive opposition to Roma* consists of respondents who object to Roma immigrants but willing to admit immigrants belonging to all other four ethno-religious groups (i.e. same race/ethnic group, different race/ethnic group, Muslims, and Jewish).

Table 3 presents the percent distribution of seven categories of respondents' attitudes. The findings reveal that two thirds of Europeans can be classified as “pro-admission.” In other words, substantial numbers of Europeans are willing to accept at least a few people from each one of the five ethno-religious groups. By contrast, only 3.4 percent of Europeans are classified as “total exclusionists.” These Europeans flatly oppose to admission of any immigrant by stating “allow none” regardless of the ethno-religious origin of the immigrant. The category of “exclusive admission” is composed of the 1.4 percent of respondents who support only admission of immigrants of the same race and ethnic group (as most country people) but oppose to admission of any person from all other groups. More than eight percent oppose admission of Roma people but willing to accept immigrants of all other groups, and about three percent exclusively oppose to admission of any Muslim immigrant but are willing to accept immigrants belonging to all other groups. Only 0.9 percent of respondents exclusively oppose immigration of people belonging to an ethnic or racial group that is different from most people in the country, but willing to admit all other groups of immigrants. The percent of people who exclusively oppose immigration of Jewish (0.2) is too small in absolute numbers (68 cases), and thus does not allow further statistical estimation^4^. Although most of the seven categories are relatively small, they have substantive meaning; and the numbers of sampled cases in these categories allow multivariate analysis that enables an evaluation of the unique sources that drive opposition to each specific group of immigrants.

**Table 3:** Total and exclusive exclusion/inclusion.

	**Percentage (valid and weighted)**	**Numbers (unweighted)**
Pro admission	67.3	17,639
Total opposition	3.4	1,174
Exclusive support for immigration of same race/ethnic group	1.4	569
Exclusive opposition to immigration of Jewish people	0.2	68
Exclusive opposition to immigration of a different race/ethnic group	0.9	234
Exclusive opposition to immigration of Muslims	2.7	785
Exclusive opposition to immigration of Gypsies	8.4	2,808

## Results: Multivariate Analysis

The multivariate analysis is aimed at predicting the various categories of attitudes toward admission of the various groups of immigrants (listed in Table 3) in order to trace, evaluate, and compare the differential sources that drive opposition toward each group of immigrants. The first stage of the multivariate analysis seeks to answer the following two questions: what are the sources that drive total opposition to immigration (regardless of ethno-religious group) and what are the sources that drive exclusive admission of immigrants of a same race/ethnic group? To answer these questions, we estimated multinomial logit regression model with a four-category dependent variable. The four categories are: (1) Pro-admission as category of comparison; (2) Exclusive admission of immigrants of a same race/ethnic group; (3) Total opposition to immigration, and (4) all other combinations of responses. The last category serves only for control purposes. Therefore, the coefficients for “all other combination of responses” category (which have no substantive meaning) are not presented. The inclusion of such category allows us to keep the total sample when estimating the different models. The estimated coefficients of the multinomial logit equation are displayed in Table 4. The coefficients in column 1 and 2 of Table 4 pertain to the effect of each variable on the relative odds of “total opposition” (i.e., “total exclusionists”) and “exclusive admission,” respectively, as compared to “pro admission.”

**Table 4:** Multinomial regression predicting odds [Exp(B)] for “total opposition” and “exclusive admission of immigrants of a same race/ethnic group” (Pro admission is category of comparison)^a^.

	**Total opposition (total exclusionists) (1)**	**Exclusive admission of immigrants of a same race/ethnic group (2)**
Age	1.01^*^	1.01^*^
Men	1.09	0.76^*^
Education	0.89^*^	0.88^*^
Insufficient income	1.43^*^	0.77^*^
Perceived economic threat	2.11^*^	1.84^*^
Perceived cultural threat	1.52^*^	1.50^*^
Fear of crime	1.19^*^	1.14^*^
Racism	1.40^*^	2.56^*^
Have a friend from different ethnic/race origin	0.41^*^	0.47^*^
Nagelkerke pseudo R-square	0.44	

a The model includes a series of dummy variables representing each country, UK is comparison category (coefficients are not presented). In addition to “include only same ethnic/race group,” total exclusionists and pro-admission, the depended variable also includes category “other combinations” for control purposes only (coefficients are not presented).

* *p < 0.05*.

The data in column 1 demonstrate that the odds of opposing immigration of all ethno-religious groups (vs. supporting admission of all of them) tend to rise with age and to decline with education (with older people being more conservative and people with high education more liberal). The odds for total opposition (total exclusionists) tend to be higher among people with insufficient income (i.e., among economically vulnerable people). Threat of competition in economic and cultural spheres and fear of crime are likely to increase odds for total opposition to immigration, with especially high effect of perceived economic threat [Exp(b) = 2.11]. In addition, odds for “total opposition” tend to increase with level of racist views as evident by the significant and positive coefficients of racism in column 1 of Table 4. By way of contrast, intergroup contact tends to decrease “total opposition.” That is, the odds for “total opposition” are twice lower among those who have a friend from a different race/ethnic group than among those who do not have such an intergroup contact.

The odds of supporting “exclusive admission” of immigrants of the same race/ethnic group (as compared to pro-admission) are displayed in column 2 of the table. The coefficients for all the predictors included in the model are statistically significant. The analysis reveals that odds for supporting exclusive admission of immigrants of the same race/ethnic group (as compared to the odds of supporting admission of all immigrants) tend to rise with respondents' level of perceived economic and cultural threats, fear of crime and racism. Note that the impact of racism on the willingness to only admit immigrants of the same race or ethnic group as most country people, but to exclude all other ethno-religious groups of immigrants [net odds are: Exp (*B*) = 2.56] is more pronounced than the impact of racism on opposing all immigrants [net odds are: Exp (*B*) = 1.40]. Intergroup contact with members of a different race/ethnic group reduces the odds for the exclusive support of admitting immigrants of the same race/ethnic groups.

The second stage of the multivariate analysis (presented in Table 5) seeks to provide answers to the following two questions: First, whether and to what extent the sources that drive opposition to immigration vary across different ethno-religious groups. Second, if such variation exists, what are the sources of opposition that are unique to each specific group of immigrants? The three groups of immigrants on which the present analysis focuses are: people of different race/ethnic group from most country people, Muslims, and Roma.

**Table 5:** Multinomial regression predicting odds [Exp(B)] of “exclusive opposition” to immigrants of different ethno-religious groups (Pro admission is category of comparison)^a^.

	**Different race/ethnic origin (1)**		**Muslims (2)**		**Roma (3)**	
	**Exclusive opposition to immigrants of a different race/ethnic group from most country people**	**Total opposition** ** to immigration**	**Exclusive opposition** ** to Muslim immigrants**	**Total opposition** ** to immigration**	**Exclusive opposition** ** to Roma immigrants**	**Total opposition** ** to immigration**
Age	1.00	1.01^*^	1.02^*^	1.01^*^	1.01^*^	1.01^*^
Men	0.83	1.10	0.86	1.10	1.09	1.05
Education	0.91^*^	0.89^*^	0.93^*^	0.89^*^	0.97^*^	0.88^*^
Insufficient income	0.93	1.46^*^	1.09	1.46^*^	0.91	1.47^*^
Perceived economic threat	1.39^*^	2.08^*^	1.25^*^	2.08^*^	1.20^*^	2.13^*^
Perceived cultural threat	1.31^*^	1.50^*^	1.30^*^	1.52^*^	1.19^*^	1.55^*^
Fear of crime	1.20^*^	1.18^*^	1.02	1.18^*^	1.13^*^	1.18^*^
Racism	1.04	1.39^*^	2.58^*^	1.38^*^	2.90^*^	1.39^*^
Have a friend from different ethnic/race origin	1.19	0.42^*^	0.72^*^	0.41^*^	0.77^*^	0.40^*^
Nagelkerke pseudo R-Square	0.44		0.43		0.44	

a The model includes a series of dummy variables representing each country, UK is comparison category (coefficients are not presented). In addition to ”include only this the group, total exclusionists and pro-admission, the depended variable also includes category “other combinations” for control purposes only (coefficients are not presented).

* p < = 0.05

To provide answers to these questions we estimated three multinomial logit equations. Equation 1 includes a dependent variable with the following four categories: (1) Exclusive opposition to immigrants of a different race/ethnic group from most country people; (2) Total opposition to immigration; (3) Pro-admission as category of comparison; (4) all other combinations of responses (the last category included only for control purposes, and its coefficients are not presented). In Equation 2 “exclusive opposition to immigrants of a different race or ethnicity” (as the first category of the dependent variable) is replaced by “exclusive opposition to Muslim immigrants.” In Equation 3, the first category of the dependent variable is “exclusive opposition to Roma immigrants.” The estimated coefficients displayed in Table 5 pertain to the impact of the independent variables on respondents' relative odds of “membership” in each category of opposition (vs. “pro-admission”).

The findings reveal that education tends to decrease the odds of opposing each one of the following three groups of immigrants: people of a different race or ethnicity, Muslims and Roma (as compared to the odds of admitting all five ethno-religious groups). By contrast, income and gender do not exert statistically significant effect on the “exclusive opposition” to each one of the three groups of immigrants. Age does not exert an effect on the “exclusive opposition” to immigrants of a different race or ethnicity, but increases “exclusive opposition” to Muslim and Roma immigrants.

Perceived economic and cultural threats tend to increase odds for exclusive opposition to each one of the three groups of immigrants: people of a different race or ethnicity, Muslims, and Roma. Fear of crime tends to increase opposition to immigrants of a different race/ethnic group and Roma immigrants, respectively, but does not exert a net effect on opposition to Muslim immigrants (the coefficient is statistically insignificant and very small). By way of contrast, racism prompts opposition to Muslim and Roma immigrants, as evident by statistically significant and sizable coefficients, Exp (*B*) = 2.58 and Exp (*B*) = 2.90, respectively. Curiously, however, racism does not exert a *net* effect on opposition to immigrants of a different race/ethnic group. While intergroup contact reduces the odds of opposing Muslims and Roma immigrants, it does not exert net effect on opposition to immigrants of a different race/ethnic group (as compared to support for admission of all immigrants).

## Discussion

The data demonstrate that level of opposition to immigration in Europe is far from being uniform and is, in fact, hierarchical, with the level of opposition being most extreme toward Muslims and Roma and quite minor toward people of the same ethnic/race groups as well as Jews. The hierarchal order is clearly reflected by the degree of overlap in opposition across groups. For example, Europeans who oppose admission of immigrants of the same race and ethnicity as the people who live in Europe are most likely to oppose admission of Roma and Muslim immigrants. However, Europeans who oppose admission of Roma and Muslim immigrants are not necessarily against admission of immigrants of the same race and ethnicity of the people who live in Europe. These findings are in line with previous research. For example, Strabac and Listhaug (2008) found that the percentage of the majority population in Europe objecting to Muslims as neighbors is higher than that objecting to immigrants (in general) as neighbors (Strabac and Listhaug, 2008). Fontanella et al. (2016, p. 487) suggest that Roma people are the most rejected ethnic group in Europe concluding that “the Roma people continue to be the most discriminated even with respect to migrants and to be classified as a separate reality to which we will not ever get used.”

Not only does the level of opposition to immigration vary across the immigrant groups but also the sources that drive opposition to immigration vary across groups. In line with general theoretical expectation, the analysis reveals that the sources of public opposition to immigration can be divided into 2 major categories: universal sources and group-specific sources. Specifically, we suggested that threats of competition and intergroup contact are universal sources while fear of crime and racism are group-specific sources. As expected, threats of competition over socio-economic resources and cultural values of society are found to be universal sources that prompt objection to immigrants regardless of their ethnic or religious origin. However, the findings do not confirm the expectation that intergroup contact reduces exclusionary attitudes toward all immigrant groups. The findings reveal that inter-group contact, similar to racism and fear of crime are, in fact, group-specific sources. Racism and lack of intergroup contact tend to prompt (exclusive) opposition to Muslim and Roma immigrants, but not to immigrants belonging to a different race/ethnic group from most country people. Fear of crime tends to prompt (exclusive) opposition to immigrants of different race/ethnic group and Roma immigrants. However, fear of crime does not appear to increase exclusive opposition to Muslims.

From theoretical point of view, the data presented here lend support to the argument that exclusionary attitudes toward immigrants are driven by multiple sources. Exclusionary views should be viewed and understood not only as a response to competitive threats posed by immigrants to the economic interests of majority population or to cultural values and homogeneity of the society but also by racist views, lack of intergroup contact and fear of crime. Whereas, threats of competition in the economic and cultural spheres increase opposition toward admission of immigrants, regardless of their specific ethnic and religious origin, racist views, lack of intergroup contacts, and fear of crime are group specific. Indeed, the findings presented here suggest that opposition to immigration as a form of anti-immigrant sentiment should be understood within a multi-dimensional framework along multiple sources that vary across the different groups of immigrants.

## Author Contributions

AG and MS: conceptualization, methodology, writing original draft and writing - review and editing; AG: statistical analysis.

### Conflict of Interest Statement

The authors declare that the research was conducted in the absence of any commercial or financial relationships that could be construed as a potential conflict of interest.

## Footnotes

^1^It is important to note, however, that previous research suggest that the public fails to accurately estimate crime rate tendencies and that fear of criminal activity by immigrants is often fuelled by anecdotal reports in the mass media (McLaren and Johnson, 2007; Rumbaut and Ewing, 2007).

^2^As a robustness check this variables was also constructed using only the two first questions that pertain to so-called biological racism. The revised operationalization of the variable did not alter the results

^3^In Portugal, Norway and Sweden as well as in Hungary, Lithuania, Slovenia and Poland, the opposition toward immigration of Jewish people is higher than that toward people of a different race and ethnicity.

^4^This category is eliminated from the multivariate analysis.
